# Incarcerated Femoral Hernia Containing Ipsilateral Fallopian Tube

**DOI:** 10.1155/2010/741915

**Published:** 2010-10-17

**Authors:** Stefanos Atmatzidis, Grigorios Chatzimavroudis, Dimitrios Dragoumis, Konstantinos Atmatzidis

**Affiliations:** ^1^“G. Gennimatas” Hospital, 2nd Surgical Clinic, Aristotle University of Thessaloniki, Ethnikis Aminis, 41, P.O. 54 635, Thessaloniki, Greece; ^2^Ioannou Michail, 7, P.O. 54 622, Thessaloniki, Greece

## Abstract

Femoral hernias are more common in women and lead to a substantial higher rate for an emergency operation, due to strangulation. Incarcerated femoral hernia with fallopian tube as a content is an extremely rare condition. A 20-year-old woman presented to the emergency department complaining of a 6-day right groin swelling, which became painful and tender to palpation during the last 48 hours. Preoperative ultrasonography detected an oedematous hernia sac, above the femoral vessels, suggesting the presence of an incarcerated femoral hernia. The patient eventually underwent emergency surgery and the diagnosis of a strangulated femoral hernia sac, containing fallopian tube, was established. No resection of the uterine tube was performed and the hernia was repaired with polypropylene plug. The postoperative period was uneventful and the woman was discharged on the second postoperative day.

## 1. Introduction

Although femoral hernia is less common than inguinal hernia, it is usually associated with incarceration and often causes significant morbidity and mortality. Femoral hernias are more common in women, but herniation of the fallopian tube in groin hernias is generally found in pediatric population. Incarcerated femoral hernia with fallopian tube as a content is an extremely uncommon entity [1]. To the best of our knowledge, there have been described three similar cases in the medical literature [2–4]. Because of its rarity, it is essential to thoroughly recognize the characteristics and content of incarcerated femoral hernia, before or even during the operation, in order to choose proper surgical management. 

The aim of this case study was to present an extremely obscure case of a right incarcerated femoral hernia, with ipsilateral fallopian tube alone, without the ovary, within the hernia sac.

## 2. Case Report

A 20-year-old woman presented to the emergency department complaining for a 6-day history of right groin swelling which gradually became tender to palpation during last 48 hours. Twenty-four hours before her admission to our department, the patient was examined by her gynaecologist, who did not find any obvious gynaecological disorder.

On examination to our department, her temperature was 37°C, pulse rate 76 beats/min, blood pressure 110/85 mmHg, and respiratory rate 16/min. Physical examination revealed a right groin mass in the femoral region, which was nonreducible and exquisitely tender to palpation. Abdominal examination also disclosed mild tenderness in the right lower quadrant. A gentle attempt to reduce the hard mass, under mild analgesia, was unsuccessful. 

**Figure 1:**
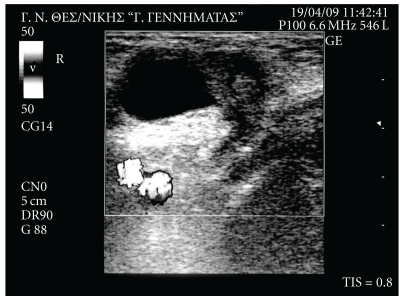
Ultrasonography showing the oedematous hernia sac, above the femoral vessels.

**Figure 2:**
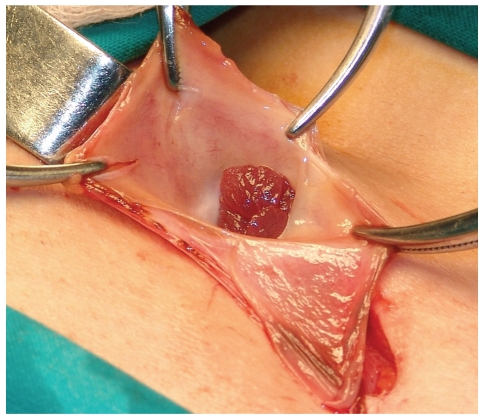
The open femoral hernia sac with its uncommon content, the ipsilateral fallopian tube.

**Figure 3:**
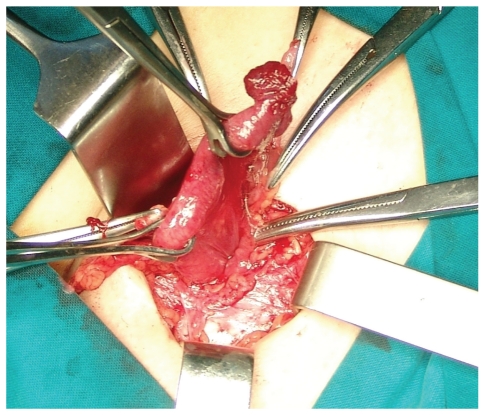
The uterine tube with its mesosalpinx, while the right ovary lies within the abdominal cavity.

The leukocyte count was 9.700/mm^3^ (Ne: 79.5%), while C-reactive protein was 0.1 mg/dl. Plain abdominal radiograph was unremarkable. Ultrasonographic examination of the right groin demonstrated a hernia sac, above the femoral vessels, measuring approximately 3.4 cm on diameter and containing suspected aperistaltic bowel segment with oedematous wall (Figure 1). Based on these findings, the diagnosis of an incarcerated femoral hernia was set and the patient underwent emergent operation. During exploration of the right groin region through femoral approach, a strangulated femoral hernia sac containing right fallopian tube was detected (Figure 2). The uterine tube was totally in the hernia sac with its mesosalpinx, while the right ovary was palpated within the abdominal cavity (Figure 3). 

No signs of ischemic damage were detected and the fallopian tube was returned to the abdominal cavity. The sac was closed at its base, the redundant portion was amputated, and the femoral hernia was repaired with polypropylene plug implantation. 

The patient had an uneventful recovery and was discharged on the second postoperative day.

## 3. Discussion

The incidence of femoral hernia is approximately 2 to 8% in adults, comprising about 30% of groin hernias. It is most commonly observed between the ages of 40 and 70, being very rare in younger ages. Femoral hernia is four-to-five times more common in females than in males and right-sided presentation is more common than left, although the reason is not well delineated in the literature [1, 5].

The aetiology of femoral hernia has been controversial throughout the years. The theory of a congenital preformed peritoneal sac in femoral hernia was advanced by many authors in the early twentieth century. McVay stated that the width of the femoral ring, which is determined by the length of the fanwise insertion of the iliopubic tract to Cooper' ligament, is the main etiologic factor of the femoral hernia.

On the other hand, Nyhus noted that acquired weakness of the transversalis fascia leads to a consequent predisposition to the development of the femoral hernia, due to increased intra-abdominal pressure. According to the most acceptable theory, the primary cause for the formation of femoral hernia is a congenitally narrow posterior inguinal wall attachment onto Cooper's ligament with a resultant enlarged femoral ring, while the secondary aetiology is a state of prolonged and increased intra-abdominal pressure, which forces preperitoneal fat into the congenitally large femoral ring [1, 5, 6]. However, it should be noted that in younger ages with femoral hernias, processes responsible for elevated intra-abdominal pressure are rarely encountered. 

The first recorded case of inguinal hernia containing uterine tube alone, without ovary, was described by Voigt in 1809 [7]. Since then, a few number of similar cases have been reported, most of which have been found in pediatric population [8]. Incarcerated femoral hernia containing fallopian tube is an extremely rare condition, as only three cases have been reported [2–4]. On the other hand, the presence of ovary in the sac of a femoral hernia is more common, with more than 10 cases having been described so far [9].

In general, the presence of fallopian tube within the sac of a groin hernia is usually associated with congenital anomalies of the genital tract. For instance, the canal of Nuck, in the younger subjects, projects as a tubular process for some distance into the inguinal canal when it is still patent, or not yet completely obliterated, and, together with the uterus, occupies a relatively high position in the abdominopelvic cavity. As a consequence, it is nearer the internal inguinal and femoral ring, and can more readily pass through these structures than in later life, when the uterus and its adnexae are, under ordinary circumstances, deeply situated in the pelvis. However, when increased intra-abdominal pressure exists, the position of the internal genital organs changes, the uterus and tubes ascend above the pelvic brim, and in this way more favourable conditions for hernia of the uterine tube are developed.

Reduction of the sac content (ovary or uterine tube) of femoral hernia should always be attempted in reproductive young woman and children without any ovarian and tubal abnormalities, provided that any life-threatening complication, such as acute salpingitis, does not exist [10, 11]. In our case, fallopian tube was preserved, since we ensured viability of the organ intraoperatively.

The preoperative diagnosis of femoral hernia is still a challenging issue. In previous reports, the diagnostic accuracy ranged from 25% to 40% [12]. Inguinal hernia, lymphadenitis, lymphangioma, lipoma, and other inguinal tumors are suggested misdiagnoses. The difficulty in diagnosis has been related to the rarity of the condition, surgeon's inexperience, the greater relative frequency of inguinal hernia, inadequate physical examination, and failure to suspect a femoral hernia, when no inguinal hernia has been identified. Femoral hernia is best visualized as a hernia in contact with the femoral vein by ultrasonography. Doppler ultrasonography might be helpful to evaluate blood flow of the containing viscera or the anatomic relation between femoral vessels and hernia sac [9, 12].

There are different surgical repair techniques for femoral hernia. McVay operation, polypropylene plug mesh technique, and laparoscopic approach are all surgical modalities that are used by surgeons today. In our patient, we applied a polypropylene plug-mesh hernioplasty technique. 

Recurrence rate after femoral hernia repair is reported to be 1%–10% [1, 5, 13, 14]. Technical insufficiency and lack of anatomical knowledge of the femoral hernia ring are considered as the most important factors for recurrence. Importantly, the introduction of polypropylene plug mesh technique has led to significant drop in the rate of recurrence [1].

From a clinical point of view, it is essential to always keep in mind that femoral hernia sac may contain almost any abdominal organ such as intestine, bladder, omentum, ovary, Meckel's diverticulum, uterus, vermiform appendix, or even fallopian tube, as in our case. For this purpose, a detailed medical history, careful physical examination, and thorough differential diagnosis are mandatory, in order to establish a definite diagnosis and proper surgical plan [15].
